# Methylation of Mercury in Earthworms and the Effect of Mercury on the Associated Bacterial Communities

**DOI:** 10.1371/journal.pone.0061215

**Published:** 2013-04-05

**Authors:** Stephan Raphael Rieder, Ivano Brunner, Otto Daniel, Bian Liu, Beat Frey

**Affiliations:** 1 Rhizosphere Processes Group, Swiss Federal Research Institute WSL, Birmensdorf, Switzerland; 2 Institute for Biogeochemistry and Pollutant Dynamics, ETH Zürich, Zürich, Switzerland; 3 Ecotoxicology Group, Agroscope Changins-Wädenswil, Wädenswil, Switzerland; 4 Medicine-Pulmonary, Allergy and Critical Care, Columbia University, New York, New York, United States; Auburn University, United States of America

## Abstract

Methylmercury compounds are very toxic for most organisms. Here, we investigated the potential of earthworms to methylate inorganic-Hg. We hypothesized that the anaerobic and nutrient-rich conditions in the digestive tracts of earthworm's promote the methylation of Hg through the action of their gut bacteria. Earthworms were either grown in sterile soils treated with an inorganic (HgCl_2_) or organic (CH_3_HgCl) Hg source, or were left untreated. After 30 days of incubation, the total-Hg and methyl-Hg concentrations in the soils, earthworms, and their casts were analyzed. The impact of Hg on the bacterial community compositions in earthworms was also studied. Tissue concentrations of methyl-Hg in earthworms grown in soils treated with inorganic-Hg were about six times higher than in earthworms grown in soils without Hg. Concentrations of methyl-Hg in the soils and earthworm casts remained at significantly lower levels suggesting that Hg was mainly methylated in the earthworms. Bacterial communities in earthworms were mostly affected by methyl-Hg treatment. Terminal-restriction fragments (T-RFs) affiliated to *Firmicutes* were sensitive to inorganic and methyl-Hg, whereas T-RFs related to *Betaproteobacteria* were tolerant to the Hg treatments. Sulphate-reducing bacteria were detected in earthworms but not in soils.

## Introduction

Mercury (Hg) is a naturally occurring metal, which is released in the environment by numerous natural and anthropogenic processes [1], [2]. Mercury is relatively stable in the atmosphere and can spread over the entire globe before returning to the earth's surface. In soil, Hg is highly immobile and accumulates in the top layer, mainly by binding to organic matter especially to thiol groups [3]. Tipping et al. [4] reported a critical limit of 3.3 mg Hg kg^−1^ organic matter corresponding to 0.13 mg Hg kg^−1^ soil. At lower concentrations, it is assumed that there are no harmful effects on soils organisms. By applying this definition, 60% of 34 natural forest soils studied in Switzerland would exceed this critical limit [5].

Methylmercury compounds (CH_3_Hg-R; methyl-Hg) are the most toxic Hg compounds for humans [6]. Methylation of Hg occurs through biotic and abiotic processes, although biotic processes are most important [7]. Sulphate-reducing bacteria (SRB) under anaerobic conditions seem to be of particular importance to methylate Hg [8]–[10]. In all SRB, enzymes that catalyze the reduction of sulphite to sulphide were found. Sulphite reductases enzymes (EC 1.8.99.3) consist of at least two polypeptides, encoded by the dissimilatory sulphite reductase genes *dsrA* and *dsrB* [11]. The presence of SRB in environmental samples is commonly analyzed by targeting the *dsrAB* genes [12]–[14].

The methylation and bioaccumulation of Hg have been well studied in aquatic ecosystems because consuming Hg-contaminated fish may lead to humans being poisoned. In contrast, studies of Hg, and in particular of methyl-Hg, in terrestrial ecosystems are rare. Over 90% of the invertebrate biomass in soils may consists of earthworms [15]. Earthworms play an important role in many soil-forming processes [16]. They also serve as a substantial food source for several higher organisms, such as birds and moles. Earthworms in forest soils are known to accumulate Hg and methyl-Hg [5], [17]. Bioaccumulation factors (BAF) of Hg in earthworms were between 1 and 15 whereas BAF for methyl-Hg ranged from 15 to 191 [5]. The lipophilic property of methyl-Hg results in more efficient bioaccumulation than inorganic-Hg, which may explain why there are considerably higher BAF for methyl-Hg than for inorganic-Hg. Another possibility for the high BAF in earthworms is, that inorganic-Hg is methylated in earthworms, e.g. due to the activity of the microbiota in their digestive tracts. In particular, unique conditions prevail in the earthworm gut, which is anaerobic, with large amounts of easily available carbon, and these may favour the anaerobic growth of microorganisms [18], [19].

In this study we tested this possibility and hypothesized that the conditions in earthworms' digestive tracts favour the methylation of Hg by their gut-inhabiting bacteria. Earthworms (*Lumbricus terrestris* L.) were either grown in sterile soils treated with mercury(II)chloride (HgCl_2_), with methylmercurychloride (CH_3_HgCl) or in soils without Hg treatment. The total-Hg (inorganic + organic Hg compounds) and methyl-Hg concentrations in soils and earthworms were analyzed after 30 days. The impact of Hg on the total bacterial community structures and compositions in earthworms were studied by molecular analyses. Because biotic Hg methylation is generally attributed to SRB, we determined the genetic potential for sulphate reduction by analysing the presence of *dsrA* genes in the bacterial communities in soils and earthworms. To the best of our knowledge, ours is the first study to investigate the ability of earthworms to methylate inorganic-Hg under natural conditions (soils).

## Materials and Methods

### Ethic statement

The research institute WSL has a general permit to use the area of their surrounding for scientific purposes. No endangered or protected species were involved in the experiment.

### Experimental design

**Figure 1:**
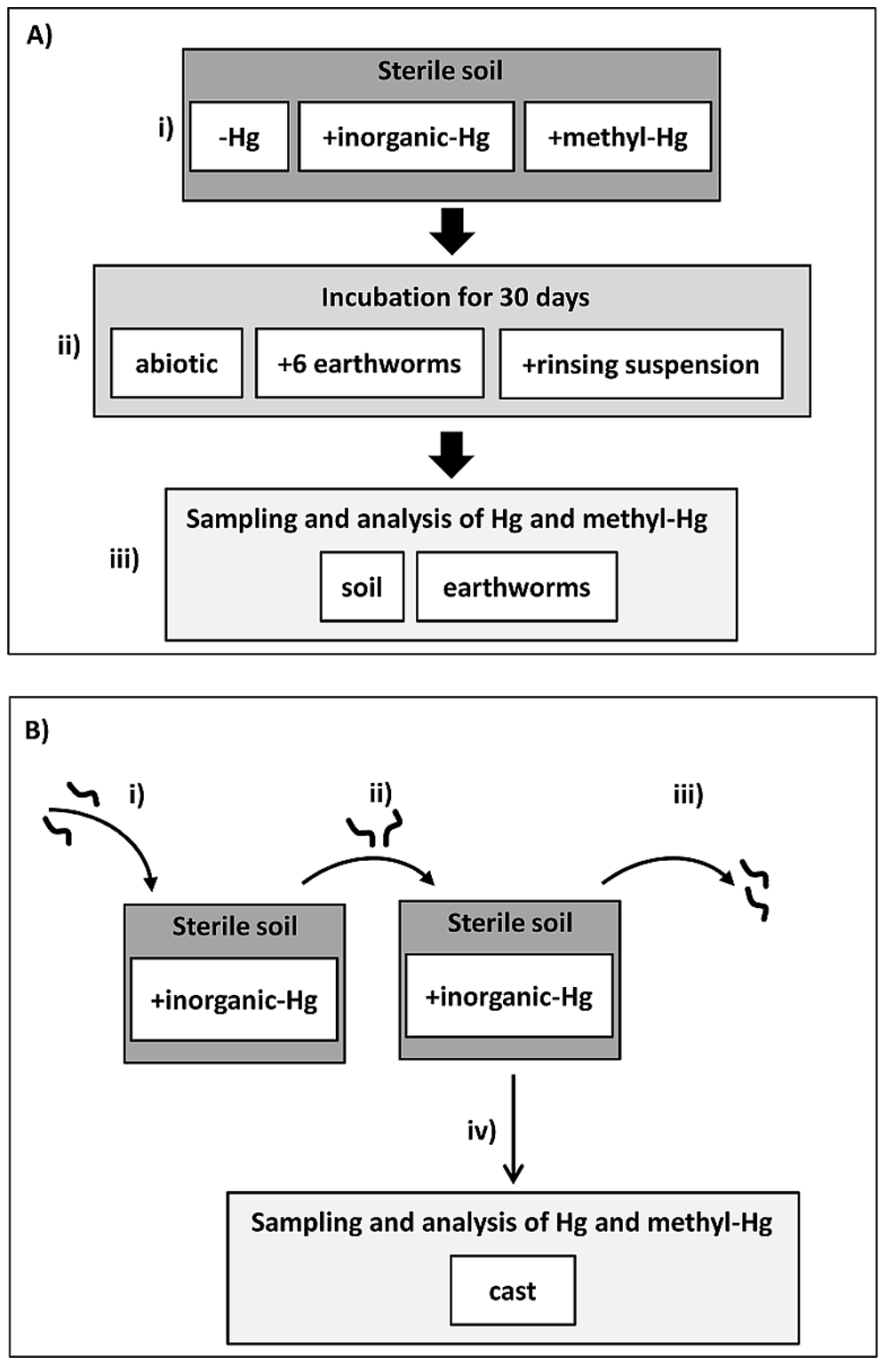
Experimental design. Two experimental assays (A and B) were performed: In the main experiment (A) sterile soil was either treated with inorganic Hg (+inorganic−Hg), methyl-Hg (+methyl−Hg) or without Hg (−Hg) (i). The soils were incubated abiotically, with earthworms or with a earthworm rinsing suspension for 30 days in the dark at 15°C (ii). At the end of incubation, the earthworms were removed from the soil and the Hg and methyl-Hg concentrations in the soil and earthworms were determined (iii). In a separate experiment (B), casts (excreted feces) were used as controls to study a possible methylation of Hg by organisms introduced into the soil by earthworms. Earthworms were incubated for one week in soils treated with inorganic-Hg (i) before they were placed into new boxes containing soils treated with Hg (ii). After three days, the earthworms were removed (iii). Immediately after removing the earthworms a cast sampling period has been started for 28 days (iv).

Laboratory experiments with *Lumbricus terrestris* L. and soils treated with inorganic-Hg, with methyl-Hg or without any Hg compounds were conducted (Figure 1). The experiments were performed in 3.3 L high density polyethylene boxes filled with 2 kg dw sterile soil. In the main experiment (Figure 1A) three approaches were used: a) soils without earthworms to examine the abiotic methylation and demethylation of Hg-species in the soil, b) soils without earthworms, but with a suspension obtained from rinsing the earthworms body surface to examine a possible Hg-methylation in the soils driven by microorganisms carried in by earthworms and c) soils with earthworms to examine the methylation of Hg in the earthworms. Sterile soil was necessary to test the ability of the microbes in the earthworm gut to methylate Hg. Otherwise, methyl-Hg in soils produced by microbes would have been taken up directly by earthworms without forming it in the gut of earthworms. Sterile soil is expected to have an impact on the "mutualistic digestive system", however, preliminary experiments showed that growth and mortality of earthworms grown in sterile soil were not affected (data not shown).

In a separate experiment, casts (excreted feces) were used as controls to study a possible methylation of Hg by organisms introduced into the soil by earthworms (Figure 1B). We analyzed earthworm cast also, because it offer very different environmental conditions for microbial growth than the surrounding soil [20].

The experimental soil was collected in a forest close to the Swiss Federal Research Institute WSL, Birmensdorf. Soil properties (pH, C/N, clay-silt-sand content) were determined according to FAL [21] before the soils were treated. The soil was dried at 105° C for two days, sieved (4 mm), homogenized and autoclaved three times. Soil aliquots were incubated for three days on petri dishes containing MMN-Agar media to test the sterility of the soils. After autoclaving, soil aliquots were pooled to three samples for analyses the initial concentrations of Hg and methyl-Hg in the soil. Table 1 summarizes the main physico-chemical properties of the soil. One part of the sterile soil was treated with HgCl_2_ [Merck (nr. 4419.0050), USA; 30 mmol resp. 6 mg Hg kg^−1^ soil; named soil+inorganic−Hg], a second with CH_3_HgCl [Sigma-Aldrich (nr. 33368), USA; 3 mmol resp. 0.75 mg methyl-Hg kg^−1^ soil; named soil+methyl−Hg] and a third was left untreated (named soil-Hg) (Figure 1A, i). The water content of each soil was adjusted to 30% by adding distilled Milli-Q water. During the experiments the water content was determined gravimetrically at each second day and the water loss was compensated by adding sterilized Milli-Q water.

**Table 1:** Soil properties and Hg and methyl-Hg concentrations (mean ± SD) in the soil used in the laboratory experiments.

Soil type	pH	C/N	Clay [%]	Silt [%]	Sand [%]	Hg tot [mg kg^−1^]	Methyl-Hg [ µg kg^−1^]
Cambisol	7.0	10.8	25	39	36	0.09±0.00	0.32±0.16

*L. terrestris* was chosen as the model organism as it is very abundant in soils of deciduous forests in Switzerland [17] and the fraction of methyl-Hg compared to the total-Hg in its tissue is high [5]. Juvenile earthworms were obtained from a commercial source (National Bait Inc., Canada). Before incubation of the earthworms in sterile soils, the earthworms were grown in the untreated collected forest soil (not sterilized, not Hg treated) for six weeks for adapting the earthworms and their gut-inhabiting bacteria to the new condition. Thereafter, the earthworms were kept in boxes with several layers of moist filter papers without feeding for 6 days at 15°C to let them empty their guts. To clean the body surface of the earthworms and to obtain a suspension of mucus, soil and microorganisms adhering to the earthworm's body surface, each individual was rinsed in a 0.8% NaCl solution. Thereafter, six earthworms were taken and pooled to three samples for analysing the initial total and methyl-Hg concentrations. The earthworms contained about 0.1 mg Hg kg^−1^ dw and 5.1 µg methyl-Hg kg^−1^ dw (4.9% methyl-Hg of total-Hg) at the beginning of the experiments.

In the experiments with earthworms, six individuals were put into each box. Equal experiments were conducted without earthworms and with a suspension obtained from earthworm surfaces (Figure 1A, ii). Freeze-dried lettuce was used as a food source, and 0.5 g was added to each box in all experiments (also in experiments without earthworms). The boxes were incubated at 15°C in the dark for 30 days. We assumed that the soil treatment should not harm the earthworms according to Ernst and Frey [22] and Lock and Janssen [23]. All experiments were performed in four replicates. The soils were sampled at the beginning and at the end of the experiments. The soil in each box was totally mixed before and after incubation and at each time point several aliquots were taken and pooled together. At the end of experiments the earthworms were starved for six days on several layers of moist filter paper until their gut contents had emptied (Figure 1A, iii). The six earthworms per each box were pooled (resulting in n = 4 per treatment), placed in liquid nitrogen and stored at −20°C until further treatment. Before chemical and microbial analyses, the frozen earthworms were lyophilized and milled.

To study and collect casts, ten earthworms were incubated as described above in soil treated with inorganic-Hg for one week (Figure 1B, i). Thereafter, the earthworms were placed into new boxes containing soil treated with inorganic-Hg (Figure 1B, ii) and after three days, they were removed (Figure 1B, iii). The earthworm-free boxes were kept in the dark with constant water content for 28 days. To observe a potential ageing effect, subsamples of earthworm casts were sampled (about 500 mg) at the day of removing earthworms (cast was excreted between 0 and 3 days ago), after 2, 7, 15 and 28 days (Figure 1B, iv). The collected samples were stored at −20°C. The samples were lyophilized, milled and stored in the dark until analysis. The cast experiment was investigated in four replicates.

### Mercury analyses

The total-Hg (inorganic + organic Hg compounds) and methyl-Hg concentrations were measured in all samples. The total-Hg concentrations in the samples were determined by using an atomic absorption spectrophotometer according to the manufacturer's instructions (Advanced Hg Analyser; AMA 254, Altec s.r.l., CZ). The methyl-Hg concentrations were determined by Gas Chromatography-Atomic Fluorescence Spectroscopy (GC-AFS) according to Liu et al. [24]. The accuracy of the total-Hg and methyl-Hg analyses was checked against certified reference materials (TORT-2 for biotic and ERM CC580 for soil samples) and its recovery ranged between 95 and 106% for Hg and between 83 and 112% for methyl-Hg.

### DNA extraction and PCR of 16S rRNA and *dsrA* genes

Genomic DNA in the earthworms and soils was extracted using a modified bead-beating method described in Frey et al. [25]. Approximately 500 mg of earthworm tissue or soil sample were processed with a BioFastPrep system (ThermoSavant). The extracted DNA was quantified with Pico Green (Invitrogen, Carlsbad, CA, USA) and stored at −20°C. DNA aliquots extracted from earthworm and soil samples (5 ng µl^−1^) were pretreated with BSA at 95°C for 4 min to remove PCR inhibitors. Bacteria in earthworms were amplified by a polymerase chain reaction (PCR) targeting the 16S rRNA genes similarly to that described in Frey et al. [26]. Twenty ng of pretreated DNA was added to 20 µl PCR reaction mix containing 1x PCR buffer, 0.5 mM MgCl2, 400 µM dNTP, 0.6 mg ml^−1^ BSA and 0.05 U µl^−1^ Hot star Taq polymerase (Qiagen), 0.2 µM of the forward primer 27F (5′-AGAGTTTGATCMTGGCTCAG-3′) and of the reverse primer 1378R (5′-CGGTGTGTACAAGGCCCGGGAACG-3′) were prepared. For T-RFLP analyses the forward primers were fluorescently (FAM) labelled. The PCR reactions were conducted in a Veriti Thermal Cycler (Applied Biosystems, Foster City, USA) and were started by an initial denaturizing step for 15 min at 95°C, followed by 35 cycles of the following steps: 95°C for 45 s, 48°C for 45 s and 72°C for 2 min. The reaction was finished by an extension step for 5 min at 72°C.

SRB were analyzed in soil and earthworm samples using for DNA amplification primers targeting the *dsrA* subunit [13] followed by agarose gel electrophoresis similar as described by Yu et al. [27]. Two separate PCR were conducted using two different reverse primers. We used the forward primer DSR1F (5′-ACS CAC TGG AAG CAC G-3′) and the reverse primers DSR4R (5′-GTG TAG CAG TTA CCG CA-3′) or DSR1334R (5′-TYT TCC ATC CAC CAR TCC-3′) described by Santillano et al. [14]. The PCR reagents were similar to those used previously for the 16S rRNA genes. After an initial step at 94°C for 15 min, 42 cycles of the following steps were performed: 94°C for 45 s, 55°C for 1 min and 72°C for 2 min. The reaction was finished at 72°C for 5 min and the PCR products were verified by agarose electrophoresis and subsequently analysed under UV illumination.

### T-RFLP profiling of total bacterial community in earthworms

16S rRNA amplicons were digested with 0.2 U *MspI* restriction enzymes according to the manufacturer's recommendations (Thermo Fischer Scientific, Waltham, USA). The digested DNA was purified with the Montage SEQ_96_ Sequencing Reaction Cleanup Kit (Millipore, Billerica, USA) according to the manufacturer's instructions. For capillary electrophoresis, 1 µl of the purified digestion product was mixed with 12.9 µl HiDi formamide (Applied Biosystems) and 0.1 µl of ROX500 DNA fragment length standard (Applied Biosystems) before heating at 95°C for 2 min. T-RFLP profiles were performed using the ABI Genetic Analyzer 310 (Applied Biosystems) and then analyzed using the software GeneScan V.3.1. and Genotyper V.2.5. (Applied Biosystems) according to Frey et al. [26]. The size and relative abundance were defined for peaks between 50 and 500 bps by applying a threshold value of 100 fluorescence units. The data for the T-RFLP analyses were standardized by calculating the relative abundance of each T-RF as described by Zumsteg et al. [28].

### Cloning and sequencing of 16S rRNA and *dsrA* genes

One clone library was generated from the 16S rRNA genes collected from earthworms to study the composition of the total bacterial communities, and one clone library was performed with *dsrA* genes from earthworms. For the clone libraries, the DNA extracted from earthworms from all Hg treatments (soil-Hg, soil+inorganic-Hg, soil+methylHg) was pooled for the PCR of 16S rRNA and *dsrA* genes separately, similar as described before except for using unlabelled primers. The PCR products were purified with Montage PCR Centrifugal Filter Devices (Millipore) and quantified using UV-VIS spectroscopy (NanoDrop Spectrophotometer ND-1000, Wilmington, USA).

The amplified and purified 16S rRNA and *dsrA* genes were cloned into pGEM-T Easy Vectors according to the manufacturer's instructions (Promega, Wisconsin, USA). We selected 400 colonies (384 for 16S rRNA genes and 16 for *dsrA* genes) and conducted PCR using vector-specific primers (M13F and M13R) as described by Frey et al. [29].

T-RFLP analysis of the colony PCRs was conducted as described above. The T-RFLP profiles of the clones were compared with the profiles of the environmental samples (earthworms). By overlapping the whole community profile with the clone profile, the clone could be assigned to a precise fragment size category. Clones yielding the selected fragments of interest (dominant T-RFs or T-RFs which were affected by the three soil Hg treatments) were sequenced. In total, 136 clones (120 clones for 16S rRNA and 16 clones for *dsrA*) were sequenced using the 3730XL DNA sequencer (Applied Biosystems) and using the forward primer 27F (5′-AGAGTTTGATCMTGGCTCAG-3′) and the reverse primer 907R (5′-CTACGGCTACCTTGTTACGA-3′) for 16S rRNA genes, or the primer SP6 (5′-ATTTAGGTGACACTATAG-3′) and T7 (5′-TAATACGACTCACTATAGGG-3′) for *dsrA* genes. The sequences were then checked and manually edited in the software BioEdit (V.7.1.3.0 by Tom Hall), chimera checked (http://comp-bio.anu.edu.au/bellerophon/bellerophon.pl) and a BLAST search was conducted in the NCBI database (http://www.ncbi.nlm.nih.gov) and the ribosomal database project (rdp.cme.msu.edu). The 16S rRNA gene sequences were deposited in Genbank under accession numbers between JX183735 and JX183794, the *dsrA* gene sequences under the accession numbers between JX461240 and JX461242.

Sequences were aligned with the ClustalW sequence alignment in BioEdit. Phylogenetic trees were calculated using Bayesian inference with the program MrBayes (ver. 3.2) [30] and the LG+I+G model. A Markov chain Monte Carlo simulation was run for 2,000,000 generations. Trees were visualized using the software FigTree (ver. 1.3.1).

### Statistical analyses

The statistical analyses of total-Hg and methyl-Hg concentrations in the samples were performed with the program STATISTICA (StatSoft, Tulsa, USA). Calculations for statistic significance (p values) of total and methyl-Hg concentrations in soils and earthworms from the different experimental approaches and in the cast experiment were performed using the Kruskal-Wallis test (p≤0.05). The effects of the three soil Hg treatments on the abundance of particular T-RFs were tested by ANOVA and Post-hoc Tukey-HSD test using STATISTICA (StatSoft). A Bray Curtis similarity matrix [31] from square-root-transformed T-RFLP data was calculated and Principal Coordinate Analysis (PCoA) was performed to estimate the Hg treatments (soil-Hg, soil+inorganic-Hg, soil+methyl-Hg) on the bacterial community structure using the software Primer 6 v.6.1.13 and Permanova v.1.0.3 (Primer-E, UK). The significance of the effects of Hg-treatments on the community structures (T-RFLP profiles) was tested by performing Permutational MANOVA analyses.

## Results

### Hg and methyl-Hg contents

After the incubation, the concentrations of total-Hg in soils were not significantly different (p<0.05) from the initial concentrations in all experiments. Before incubation, the methyl-Hg concentration in soils treated with methyl-Hg was similar as the total Hg concentration (data not shown). The concentrations of total-Hg and methyl-Hg in soils at the end of the experiments with and without an earthworm rinsing suspension (abiotic experiments) were similar. However, the concentrations of methyl-Hg in soils treated with methyl-Hg decreased by about 60% during the experiments (data not shown).

The concentrations of total-Hg and methyl-Hg in the earthworms increased in all experiments (Table 2). The initial concentrations of methyl-Hg in earthworms was about 5 µg methyl-Hg kg^−1^ soil dw. At the end of the experiments, the concentrations of methyl-Hg in earthworms were significantly higher in experiments with inorganic-Hg (75 µg methyl-Hg kg^−1^ soil dw) and with methyl-Hg (16 613 µg methyl-Hg kg^−1^ soil dw) than without Hg (11 µg methyl-Hg kg^−1^ soil dw) (p = 0.035 and 0.001 respectively; Table 2). The concentrations of total-Hg in the casts were about 25% lower than in the surrounding soils, whereas the concentrations of methyl-Hg in the casts were similar to the concentrations in the soils but did not change significantly (p<0.05) over time (Table 2).

**Table 2:** Total-Hg and methyl-Hg concentrations (mean ± SD; n = 4) in soils, earthworms and in casts.

Soil*		Hg tot [ µg kg^−1^]	Methyl-Hg [ µg kg^−1^]
Treatment	earthworms		
−Hg	without	101±5.0	0.31±0.06
−Hg	with	92±0.2	0.34±0.04
+inorganic-Hg	without	6 145±547	0.81±0.26
+inorganic-Hg	with	5 640±401	1.26±0.89
+inorganic-Hg	with rinsing suspension	6 035±523	1.00±0.55**
+methyl-Hg	without	700±18	250±37
+methyl-Hg	with	594±52	301±267
+methyl-Hg	with rinsing suspension	682±4	288±164

Earthworms*			
Treatment			
−Hg		218±57	11±2
+inorganic-Hg		24 271±5 531	75±35
+methyl-Hg		15 582±3 085	16 613±7 770

Casts***	Age [d]		
	0–3	5 248±864	1.0±0.2
	2–5	4 563±615	1.5±0.3
	7–10	4 630±622	1.2±0.5
	15–18	4 165±821	1.2±0.8
	28–31	4 619±149	0.9±0.4

* Sampled after 30 days of incubation;

** n = 3;

*** Soil treated with inorganic-Hg.

### Bacterial community profiling

**Figure 2:**
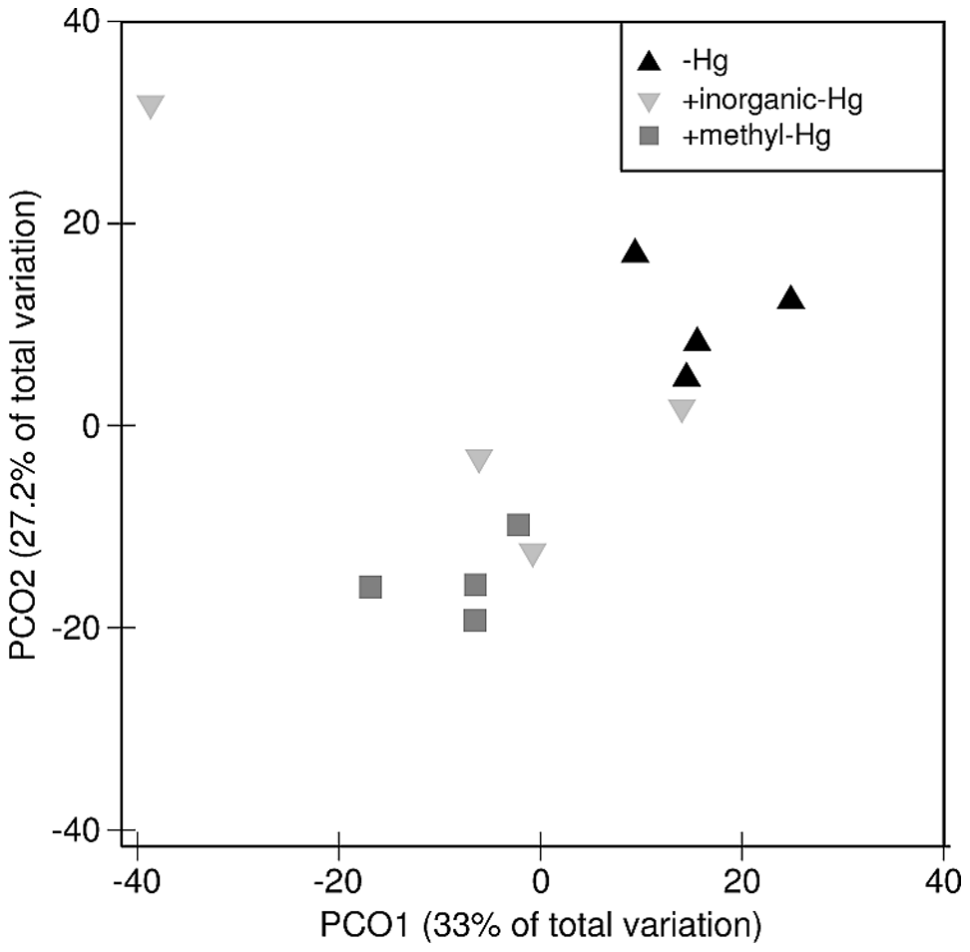
PCoA of bacterial T-RFLP profiles in earthworms. The influence of the Hg treatment (symbols) was estimated with Permutational MANOVA.

The bacterial community structures in the earthworms were strongly influenced by the Hg treatments (Table 3; Figure 2). Bacterial T-RFLP profiles in earthworms grown in soils not treated with Hg are marginally significantly different from those in earthworms grown in soils treated with inorganic Hg (p = 0.06; Permutational MANOVA) and were significantly different to those in earthworms from soil treated with methyl-Hg (p = 0.03; Permutational MANOVA). The T-RFLP profiles between the inorganic and methyl-Hg treatments were different but not on a significant level (p = 0.12). The numbers of T-RFs in earthworms grown in soil treated with methyl-Hg were lower (26±5) as in experiments with Hg (28±5) and without Hg (41±6; data not shown). Eight T-RFs (11%) in earthworms were significantly decreased in experiments with inorganic-Hg compared to experiments without Hg (Table 3). In methyl-Hg treated soils, eleven T-RFs (16%) in earthworms were significantly less and two T-RFs (3%) significantly more abundant compared to experiments without Hg (Table 3). Overall, the bacterial T-RFLP profiles in earthworms grown in soils without Hg were clearly different from those in earthworms grown in soils with methyl-Hg, according to PCoA analyses (Figure 2).

**Table 3:** Numbers and percentages of total numbers of T-RFs (in parentheses) in earthworms after 30 days which were significantly different (decreasing or increasing) between the soil treatments (−Hg, +inorganic-Hg; +methyl-Hg).

	Decreased	Increased
−Hg versus +inorganic-Hg	8 (11)	0 (0)
−Hg versus +methyl-Hg	11 (16)	2 (3)
+inorganic-Hg versus +methyl-Hg	0 (0)	2 (3)

Total number of T-RFs in earthworms: n = 71.

### Bacterial community composition

**Figure 3:**
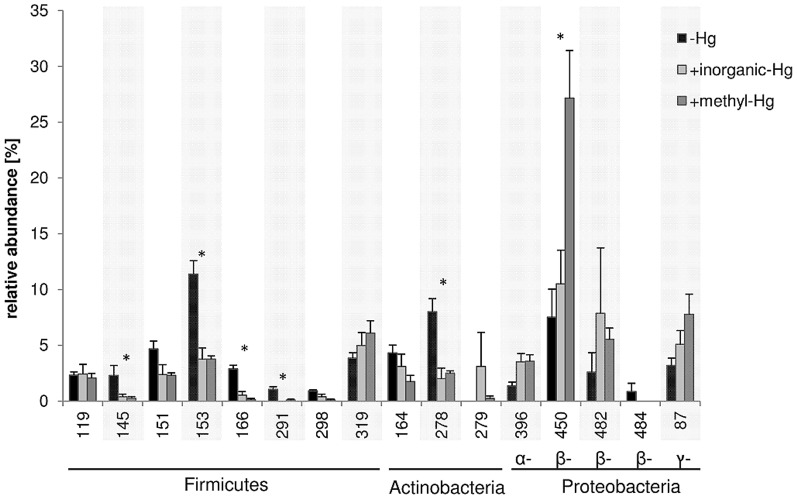
Percentage of particular bacterial T-RFs compared to the total abundance of T-RFs in earthworms. The T-RFs shown were chosen because they were (i) relatively abundant or (ii) sensitive or tolerant to Hg and methyl-Hg amendment. (*) indicates the T-RFs differed significantly (p<0.05) between the soil Hg treatments. The relationships between the corresponding clone T-RFs and the environmental sample T-RFs are shown in the Figure 4.

**Figure 4:**
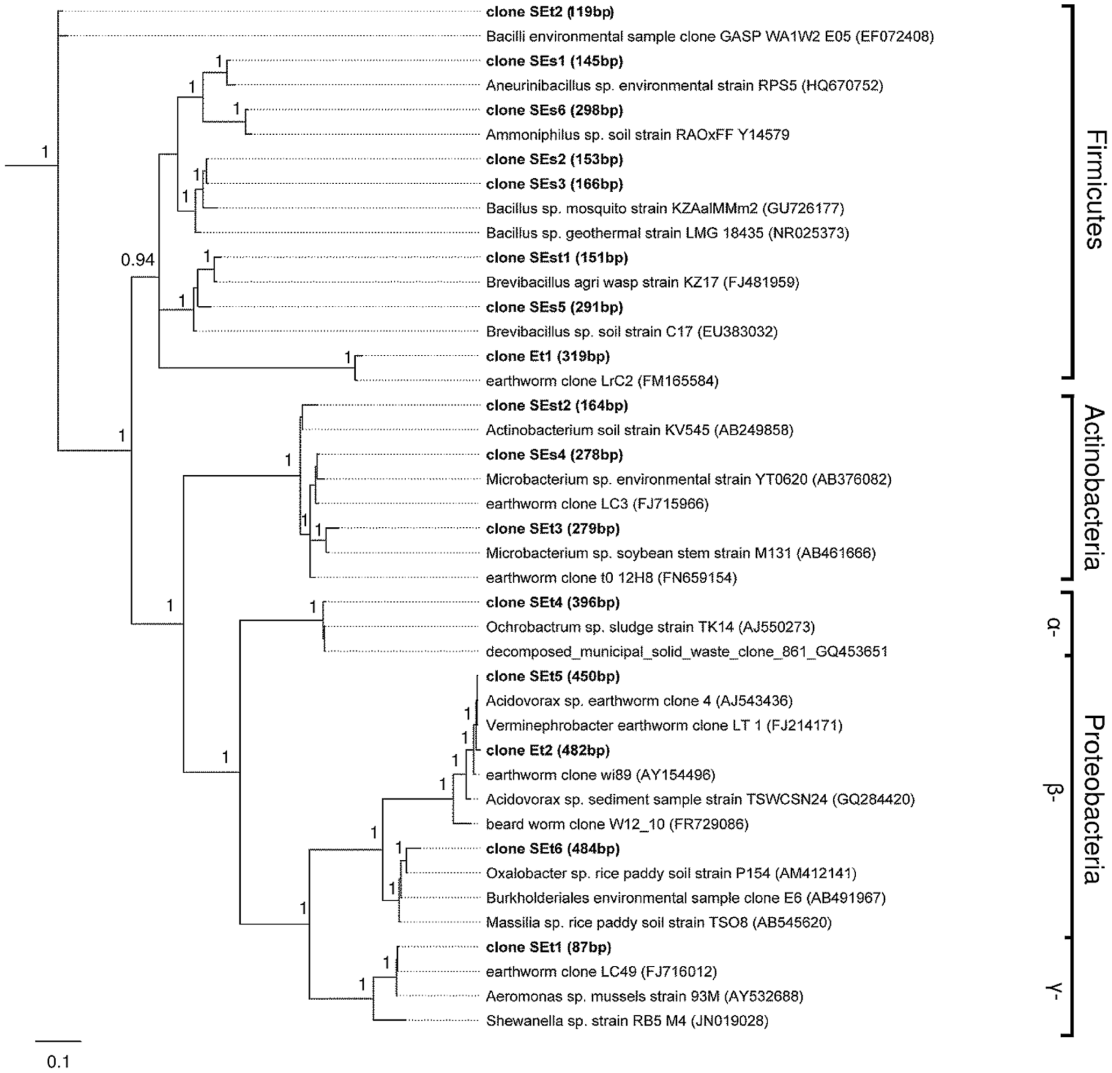
Phylogram with the phylogenetic relationships of 16S rRNA gene sequences. The phylogenetic tree shows the bacterial strains and environmental clones most closely to the T-RFs in Figure 3. Whenever possible, closest strains were used for the calculation of the tree but when no strain was available (e.g. many species of *Mollicutes* could not be isolated so far) the closest clone was used. The tree was calculated by Baysian inference using sequences of 898 bp lengths and shows the affiliation between the clones and closest related sequences of NCBI. The clones of our study are bold marked. Only bootstrap values above 0.9 are given. The scale bar represents 0.1 (10%) of sequence divergence.

The T-RF profiles of the earthworm samples were compared to the T-RF profiles of the clones. At least five clones yielding the selected fragments of interest (dominant T-RFs or T-RFs which were affected by the three soil Hg treatments; Figure 3) were sequenced. Sequence analysis confirmed that cloned sequences were similar and always matched the same closest relatives. The cloned sequences were related to the phyla *Firmicutes*, *Actinobacteria* and *Proteobacteria* (Figure 4). Eight T-RFs (119 bp, 145 bp, 151 bp, 153 bp, 166 bp, 291 bp, 298 bp and 319 bp) belong to the phylum *Firmicutes*. The cloned sequence of T-RF 151 bp (clone SEst1) was most similar (99%) to a *Brevibacillus sp*. strain KZ17 (FJ481959) isolated from wasps. In earthworms, the abundance of this T-RF (151 bp) tended to decrease in experiments with inorganic-Hg and methyl-Hg (Figure 3). Cloned sequences of the T-RFs 153 bp (clone SEs2) and 166 bp (clone SEs3) were closely related to each other, and were most similar (97%) to a *Bacillus sp*. strain KZ_AaIM_Mm2 (GU726177) isolated from mosquitoes. The abundance of both T-RFs in earthworm samples was significantly lower in experiments with inorganic-Hg and methyl-Hg than in those without Hg. Overall, T-RFs affiliated to the phylum *Firmicutes* seem to be sensitive to inorganic-Hg and methyl-Hg.

Cloned sequences of three T-RFs (164 bp, 278 bp and 279 bp) are phylogenetically affiliated to *Actinobacteria* (Figure 4). Cloned sequences of T-RF 278 bp (clone SEs4) were most similar (98%) to a *Microbacterium sp*. strain YT0620 (AB376082). T-RF 278 bp was significantly more abundant in earthworms grown in soils without Hg than in soils treated with inorganic-Hg or methyl-Hg.

We found four T-RFs belonging to *Alphaproteobacteria* (396 bp), *Betaproteobacteria* (450 bp, 482 bp) or *Gammaproteobacteria* (87 bp) that were more abundant in earthworms from soils treated with inorganic-Hg or methyl-Hg than in soils without Hg. The T-RF of 450 bp was of special interest because it was clearly more abundant in earthworms grown in soils treated with methyl-Hg, where it was the most dominant T-RF, than in soils without Hg or with inorganic-Hg. Cloned sequences of T-RF 450 bp (clone SEst3) were most similar (99%–100%) to an environmental clone (AJ543436) affiliated to *Acidovorax sp*. and to a clone LT 1 (FJ214171) related to *Verminephrobacter sp*. which were both obtained from earthworms.

We also tested the DNA extracts obtained from earthworm and also from soils to detect the presence of SRB with specific PCRs targeting the *dsrA* genes. In all earthworm but not in soil samples (−Hg, +inorganic-Hg, +methyl-Hg) weak PCR products appeared (data not shown). Cloned sequences were most similar to the *dsrA* subunit of *Desulfovibrio vulgaris* strain RCH1 (similarity between 97 and 99%; CP002297).

## Discussion

This study demonstrates that earthworms provide suitable conditions for the methylation of inorganic-Hg. In the biotic experiments with earthworms, the concentrations of methyl-Hg in soils are expected to decrease with time due to the strong uptake and retention of methyl-Hg in earthworms, and due to the decrease of methyl-Hg concentrations in soils measured in experiments without earthworms. Therefore, in these experiments, the concentrations of methyl-Hg in the soils should decrease (initial soil concentration minus loss due to accumulation by earthworms and evasion/demethylation of methyl-Hg) during incubation. This was, however, not observed here. Instead, the concentrations of methyl-Hg in soils were slightly higher (1.2 µg methyl-Hg kg^−1^ soil dw) at the end of the experiments. Hinton and Veiga [32] showed that earthworms are potentially able to methylate Hg. They dissolved Hg^0^ in tannic acid, mixed it with silica sand and paper substrate, and let earthworms (*Eisenia foetida*) grow in it. They analyzed the methyl-Hg concentrations and found a ratio of methyl-Hg to total-Hg up to 160 times higher in earthworm tissues than in the acid and the substrate. In our study, we have chosen, in contrast, a natural habitat (soil) for the earthworms and Hg^2+^, the most dominant Hg species in natural soils [33]. We were able to show, that inorganic-Hg is methylated in the earthworm itself, and not by the bacteria introduced into the soils by the earthworms. This finding is based on the following evidence. Firstly, the earthworms contained about six times higher concentrations of methyl-Hg if they grew in soils treated with inorganic-Hg than in soils without Hg. Secondly, the concentrations of methyl-Hg in earthworm casts were similar to the concentrations in the soils and did not change over time. Thirdly, the earthworm rinsing solution did not enhance the methyl-Hg concentrations in the inorganic-Hg treated soils and thus, microorganisms introduced into the soil (cast/rinsing solution) did not methylate Hg in the soils. Uncertainties of the measurements of methyl-Hg occur, since it renders difficult to separate chromatographically methyl-Hg from inorganic-Hg in samples with very high Hg concentrations. Another uncertainty is that methyl-Hg can be formed artificially during the analytical procedure as pointed out in [34]. Despite these uncertainties we were able to demonstrate evidence that inorganic-Hg is methylated in earthworms itself, and not by the bacteria introduced into the soils by earthworms.

Mercury methylation has been observed in terrestrial invertebrates. Limper et al. [35] tested the ability of termites (*Mastotermes darwinesis*) to methylate inorganic-Hg. They found an *in vivo* methylation of Hg in termites and identified SRB (*Desulfovibrio intestinalis*) in their intestinal tract as important players in the formation of methyl-Hg. In our study, SRB (*D. vulgaris*) were also detected in earthworms but not in soils. Therefore, we cannot exclude that SRB may have played a role in the methylation of the Hg in our study. *Desulfovibrio vulgaris* has also recently been shown to facilitate the methylation of inorganic-Hg in freshwater sediments [7].

The occurrence of methylation in earthworms has potentially implications for the Hg cycle in soil ecosystems as it enriches the methyl-Hg pool in soils, and consequently also in the food web. In addition, biomethylation is not only restricted to anaerobic conditions in soils as usually reported [8], [9], but also to well-drained soils.

The impact of inorganic-Hg in soils on soil bacterial community structures has already been reported [4], [36]–[41]. It is also well known, that Hg pollution changes the gut bacterial community in soil invertebrate [42]–[44]. However, as far as we know, the effects of methyl-Hg on the bacterial community structures in soils and earthworms have never been studied. Here, we showed that the bacterial community structures in the earthworms were clearly affected by Hg. Several T-RFs (87 bp, 278 bp, 279 bp, 319 bp, 450 bp and 482 bp) in the earthworms were affected by Hg and these cloned sequences had closest relatives to sequences retrieved from intestine tracts of earthworms [45]–[49]. T-RF 319 bp (clone Et1) was slightly increased with inorganic and methyl-Hg and cloned sequences were phylogenetically affiliated to *Mollicutes* obtained from earthworms. *Mollicutes* within the *Firmicutes* contain no cell walls and contain a reduced genome size as a consequence of a reductive or degenerative evolution process [50]–[52]. They obtain nutrients from their host cells (earthworm) parasitically. Therefore, the higher tolerance of *Mollicutes* to Hg could be due to the lower number of genes and enzymes affected by Hg, and/or their supply with nutrients from host cells. When the earthworms were grown in soils with methyl-Hg, T-RF 450 bp became dominant (increase from about 7% to 27% of the total abundance). The cloned sequence of T-RF 450 bp (clone SEst3) was closely related to sequences affiliated to *Acidovorax sp*. (AJ543436) and *Verminephrobacter sp*. (FJ214171) within the *Betaproteobacteria* retrieved from earthworms. *Verminephrobacter* are *Gram*-negative bacteria symbiotically colonizing the nephridia of the earthworms *Eisenia foetida*. Interestingly, they have been described as obligate aerobic organisms occurring under low oxygen conditions [53]. In contrast, *Acidovorax sp*. are able to grow outside of earthworms [53]–[55]. Nephridia are the paired excretion organs of invertebrates and are comparable to the kidneys of vertebrates. It was suggested a long time ago that nephridia are symbiotically colonized by bacteria [48], [56], but the functions of the associated bacteria are not well studied. These symbionts promote the degradation of proteins [56]. *Verminephrobacter sp*. may also play a role in the detoxification of inorganic-Hg or methyl-Hg in our earthworms. A wide range of bacteria belonging to the phyla of *Firmicutes*, *Actinobacteria*, and *Proteobacteria* are known to be Hg resistant [57], [58]. Bacterial resistance to Hg is mainly associated with the presence of mercury resistance (*mer*) operons [59]. The mercuric reductase enzyme (*merA*) catalyzes the conversion of Hg(II) to the volatile Hg(0). The *merB* enzyme degrades organic Hg compounds to the less toxic form Hg(II) [60]. However, *mer* genes are predominant in aerobic environments and were rarely found in obligate anaerobes [61] assuming that this mechanism is hardly important for this study. Other unknown mechanisms for Hg(II) reduction are suggested under anaerobic conditions [62], [63].

Another mechanism for microbial Hg resistance is based on the methylation of Hg(II) [64]. Methyl-Hg-chloride may diffuse through the cell membrane, and as a result the concentrations of Hg in the organisms probably decrease. However, the mechanisms which could be important in this study are not known and need to be further investigated.

In our study, the concentrations of methyl-Hg in soils at the start and after incubation did not vary significantly (p<0.05) in experiments with or without inorganic-Hg. Interestingly, in all soils treated with methyl-Hg, the concentrations of methyl-Hg decreased (initial: 0.75 mg methyl-Hg kg^−1^ soil dw and end: almost 0.3 mg methyl-Hg soil dw) by about 60% during the experiments (data not shown). Bacteria are able to demethylate Hg-species [60], [65], [66]. Abiotic decomposition of methyl-Hg due to photodegradation has also been reported in surface water [67]. In the abiotic experiments with sterile soil treated with methyl-Hg, the concentrations of methyl-Hg decreased by about 60%, even though the boxes were stored in the dark so that a photodegradation of methyl-Hg in soils could be excluded. Likewise, the concentrations of total-Hg in these soils did not decrease, which indicates that methyl-Hg in the soil was degraded by other factors than photodegradation.

## Conclusion

We have been able to show that the gut of earthworms provides suitable conditions for the methylation of inorganic-Hg. Control experiments (abiotic; with earthworms rinsing suspension; cast) strongly supported our finding that inorganic-Hg is methylated in earthworms itself, and not by the bacteria introduced into the soils by earthworms. SRB may play a role in the methylation of inorganic-Hg in our earthworms because they were found only in the earthworms and not in the soils. The transformation of inorganic-Hg to the much more toxic form of methyl-Hg may not only occur under anaerobic soil conditions, but also in the anaerobic guts of earthworms inhabiting aerobic soil environments. The occurrence of biomethylation in earthworms may have implications for the Hg cycle in soil ecosystems. This process may enrich the methyl-Hg pool in soils and, consequently, also may enrich methyl-Hg in the food web.

## References

[1] Schroeder WH, Munthe J (1998) Atmospheric mercury-An overview. Atmos Environ 32: 809–822.

[2] Swain EB, Jakus PM, Rice G, Lupi F, Maxson PA, et al (2007) Socioeconomic consequences of mercury use and pollution. Ambio 36: 45–61. 17408190 10.1579/0044-7447(2007)36[45:scomua]2.0.co;2

[3] Skyllberg U, Drott A (2010) Competition between disordered iron sulfide and natural organic matter associated thiols for mercury(II)-an EXAFS study. Environ Sci Technol 44: 1254–1259. 20099882 10.1021/es902091w

[4] Tipping E, Lofts S, Hooper H, Frey B, Spurgeon D, et al (2010) Critical Limits for Hg(II) in soils, derived from chronic toxicity data. Environ Pollut 158: 2465–2471. 20434245 10.1016/j.envpol.2010.03.027

[5] Rieder SR, Brunner I, Horvat M, Jacobs A, Frey B (2011) Accumulation of mercury and methylmercury by mushrooms and earthworms from forest soils. Environ Pollut 159: 2861–2869. 21621314 10.1016/j.envpol.2011.04.040

[6] Clarkson TW, Magos L (2006) The toxicology of mercury and its chemical compounds. Crit Rev Toxicol 36: 609–662. 16973445 10.1080/10408440600845619

[7] Shao DD, Kang Y, Wu SC, Wong MH (2012) Effects of sulfate reducing bacteria and sulfate concentrations on mercury methylation in freshwater sediments. Sci Total Environ 424: 331–336. 22444059 10.1016/j.scitotenv.2011.09.042

[8] Holloway JM, Goldhaber MB, Scow KM, Drenovsky RE (2009) Spatial and seasonal variations in mercury methylation and microbial community structure in a historic mercury mining area, Yolo County, California. Chem Geol 267: 85–95.

[9] Drott A, Lambertsson L, Bjorn E, Skyllberg U (2007) Importance of dissolved neutral mercury sulfides for methyl mercury production in contaminated sediments. Environ Sci Technol 41: 2270–2276. 17438774 10.1021/es061724z

[10] Barkay T, Wagner-Döbler I (2005) Microbial transformations of mercury: potentials, challenges, and achievements in controlling mercury toxicity in the environment. In: Allen I. Laskin JWB, Geoffrey MG, editors. Advances in Applied Microbiology: Academic Press. pp. 1–52. 10.1016/S0065-2164(05)57001-1 16002008

[11] Klein M, Friedrich M, Roger AJ, Hugenholtz P, Fishbain S, et al (2001) Multiple lateral transfers of dissimilatory sulfite reductase genes between major lineages of sulfate-reducing prokaryotes. J Bacteriol 183: 6028–6035. 11567003 10.1128/JB.183.20.6028-6035.2001 PMC99682

[12] Wagner M, Loy A, Klein M, Lee N, Ramsing NB, et al (2005) Functional marker genes for identification of sulfate-reducing prokaryotes. Methods Enzymol 397: 469–489. 16260310 10.1016/S0076-6879(05)97029-8

[13] Dhillon A, Teske A, Dillon J, Stahl DA, Sogin ML (2003) Molecular characterization of sulfate-reducing bacteria in the Guaymas Basin. Appl Environ Microb 69: 2765–2772. 10.1128/AEM.69.5.2765-2772.2003 PMC154542 12732547

[14] Santillano D, Boetius A, Ramette A (2010) Improved *dsrA*-based terminal restriction fragment length polymorphism analysis of sulfate-reducing bacteria. Appl Environ Microb 76: 5308–5311. 10.1128/AEM.03004-09 PMC2916486 20543035

[15] Ireland MP (1983) Heavy metal uptake and tissue distribution in earthworms. In: Satchell JE, editor. Earthworm Ecology: Springer Netherlands. pp. 247–265.

[16] Brown G (1995) How do earthworms affect microfloral and faunal community diversity? Plant Soil 170: 209–231.

[17] Ernst G, Zimmermann S, Christie P, Frey B (2008) Mercury, cadmium and lead concentrations in different ecophysiological groups of earthworms in forest soils. Environ Pollut 156: 1304–1313. 18400348 10.1016/j.envpol.2008.03.002

[18] Drake HL, Horn MA (2007) As the worm turns: the earthworm gut as a transient habitat for soil microbial biomes. Annu Rev Microbiol 61: 169–189. 17506687 10.1146/annurev.micro.61.080706.093139

[19] Horn MA, Schramm A, Drake HL (2003) The earthworm gut: an ideal habitat for ingested N2O-producing microorganisms. Appl Environ Microb 69: 1662–1669. 10.1128/AEM.69.3.1662-1669.2003 PMC150078 12620857

[20] Kizilkaya R (2004) Cu and Zn accumulation in earthworm *Lumbricus terrestris* L. in sewage sludge amended soil and fractions of Cu and Zn in casts and surrounding soil. Ecol Eng 22: 141–151.

[21] FAL (1997) Kartieren und Beurteilen von Landwirtschaftsböden. Schriftenreihe FAL, no. 24. Zurich.

[22] Ernst G, Frey B (2007) The effect of feeding behavior on Hg accumulation in the ecophysiologically different earthworms *Lumbricus terrestris* and *Octolaseon cyaneum*: A microcosm experiment. Soil Biol Biochem 39: 386–390.

[23] Lock K, Janssen CR (2001) Ecotoxicity of mercury to *Eisenia fetida*, *Enchytraeus albidus* and *Folsomia candida* . Biol Fert Soils 34: 219–221.

[24] Liu B, Yan HY, Wang CP, Li QH, Guedron S, et al (2012) Insights into low fish mercury bioaccumulation in a mercury-contaminated reservoir, Guizhou, China. Environ Pollut 160: 109–117. 22035933 10.1016/j.envpol.2011.09.023

[25] Frey B, Pesaro M, Rudt A, Widmer F (2008) Resilience of the rhizosphere *Pseudomonas* and ammonia-oxidizing bacterial populations during phytoextraction of heavy metal polluted soil with poplar. Environ Microbiol 10: 1433–1449. 18279346 10.1111/j.1462-2920.2007.01556.x

[26] Frey B, Stemmer M, Widmer F, Luster J, Sperisen C (2006) Microbial activity and community structure of a soil after heavy metal contamination in a model forest ecosystem. Soil Biol Biochem 38: 1745–1756.

[27] Yu R-Q, Adatto I, Montesdeoca MR, Driscoll CT, Hines ME, et al (2010) Mercury methylation in *Sphagnum* moss mats and its association with sulfate-reducing bacteria in an acidic Adirondack forest lake wetland. FEMS Microbiol Ecol 74: 655–668. 20955196 10.1111/j.1574-6941.2010.00978.x

[28] Zumsteg A, Luster J, Goransson H, Smittenberg RH, Brunner I, et al (2012) Bacterial, archaeal and fungal succession in the forefield of a receding glacier. Microb Ecol 63: 552–564. 22159526 10.1007/s00248-011-9991-8

[29] Frey B, Niklaus PA, Kremer J, Luscher P, Zimmermann S (2011) Heavy-machinery traffic impacts methane emissions as well as methanogen abundance and community structure in oxic forest soils. Appl Environ Microb 77: 6060–6068. 10.1128/AEM.05206-11 PMC3165404 21742929

[30] Huelsenbeck JP, Ronquist F (2001) MRBAYES: Bayesian inference of phylogenetic trees. Bioinformatics 17: 754–755. 11524383 10.1093/bioinformatics/17.8.754

[31] Bray JR, Curtis JT (1957) An ordination of the upland forest communities of southern wisconsin. Ecol Monogr 27: 326–349.

[32] Hinton JJ, Veiga MM (2002) Earthworms as bioindicators of mercury pollution from mining and other industrial activities. Geochem Explor Env A 2: 269–274.

[33] Morel FMM, Kraepiel AML, Amyot M (1998) The chemical cycle and bioaccumulation of mercury. Annu Rev Ecol Syst 29: 543–566.

[34] Nevado JJB, Martin-Doimeadios RCR, Bernardo FJG, Moreno MJ (2008) Determination of monomethylmercury in low- and high-polluted sediments by microwave extraction and gas chromatography with atomic fluorescence detection. Anal Chim Acta 608: 30–37. 18206991 10.1016/j.aca.2007.12.001

[35] Limper U, Knopf B, Konig H (2008) Production of methyl mercury in the gut of the Australian termite *Mastotermes darwiniensis* . J Appl Entomol 132: 168–176.

[36] Rasmussen LD, Sorensen SJ (2001) Effects of mercury contamination on the culturable heterotrophic, functional and genetic diversity of the bacterial community in soil. FEMS Microbiol Ecol 36: 1–9. 11377768 10.1111/j.1574-6941.2001.tb00820.x

[37] Holtze MS, Ekelund F, Rasmussen LD, Jacobsen CS, Johnsen K (2003) Prey-predator dynamics in communities of culturable soil bacteria and protozoa: differential effects of mercury. Soil Biol Biochem 35: 1175–1181.

[38] Ranjard L, Lignier L, Chaussod R (2006) Cumulative effects of short-term polymetal contamination on soil bacterial community structure. Appl Environ Microb 72: 1684–1687. 10.1128/AEM.72.2.1684-1687.2006 PMC1392981 16461728

[39] Philippot L, Cregut M, Cheneby D, Bressan M, Dequiet S, et al (2008) Effect of primary mild stresses on resilience and resistance of the nitrate reducer community to a subsequent severe stress. FEMS Microbiol Lett 285: 51–57. 18507685 10.1111/j.1574-6968.2008.01210.x

[40] Vishnivetskaya TA, Mosher JJ, Palumbo AV, Yang ZK, Podar M, et al (2011) Mercury and Other Heavy Metals Influence Bacterial Community Structure in Contaminated Tennessee Streams. Appl Environ Microb 77: 302–311. 10.1128/AEM.01715-10 PMC3019708 21057024

[41] Mosher JJ, Vishnivetskaya TA, Elias DA, Podar M, Brooks SC, et al (2012) Characterization of the Deltaproteobacteria in contaminated and uncontaminated stream sediments and identification of potential mercury methylators. Aquat Microb Ecol 66: 271–282.

[42] Lapanje A, Drobne D, Nolde N, Valant J, Muscet B, et al (2008) Long-term Hg pollution induced Hg tolerance in the terrestrial isopod *Porcellio scaber* (Isopoda, Crustacea). Environ Pollut 153: 537–547. 17988772 10.1016/j.envpol.2007.09.016

[43] Lapanje A, Rupnik M, Drobne D (2007) Gut bacterial community structure (*Porcellio scaber*, Isopoda, Crustacea) as a measure of community level response to long-term and short-term metal pollution. Environ Toxicol Chem 26: 755–763. 17447561 10.1897/06-099r.1

[44] Lapanje A, Zrimec A, Drobne D, Rupnik M (2010) Long-term Hg pollution-induced structural shifts of bacterial community in the terrestrial isopod (*Porcellio scaber*) gut. Environ Pollut 158: 3186–3193. 20724045 10.1016/j.envpol.2010.07.001

[45] Wüst PK, Horn MA, Drake HL (2011) *Clostridiaceae* and *Enterobacteriaceae* as active fermenters in earthworm gut content. ISME J 5: 92–106. 20613788 10.1038/ismej.2010.99 PMC3105676

[46] Knapp BA, Podmirseg SM, Seeber J, Meyer E, Insam H (2009) Diet-related composition of the gut microbiota of *Lumbricus rubellus* as revealed by a molecular fingerprinting technique and cloning. Soil Biol Biochem 41: 2299–2307.

[47] Nechitaylo TY, Timmis KN, Golyshin PN (2009) '*Candidatus Lumbricincola*', a novel lineage of uncultured *Mollicutes* from earthworms of family *Lumbricidae* . Environ Microbiol 11: 1016–1026. 19396950 10.1111/j.1462-2920.2008.01837.x

[48] Schramm A, Davidson SK, Dodsworth JA, Drake HL, Stahl DA, et al (2003) *Acidovorax*-like symbionts in the nephridia of earthworms. Environ Microbiol 5: 804–809. 12919416 10.1046/j.1462-2920.2003.00474.x

[49] Singleton DR, Hendrix PF, Coleman DC, Whitman WB (2003) Identification of uncultured bacteria tightly associated with the intestine of the earthworm *Lumbricus rubellus* (*Lumbricidae*; *Oligochaeta*). Soil Biol Biochem 35: 1547–1555.

[50] Pollack JD, Williams MV, McElhaney RN (1997) The comparative metabolism of the *Mollicutes* (*Mycoplasmas*): The utility for taxonomic classification and the relationship of putative gene annotation and phylogeny to enzymatic function in the smallest free-living cells. Crit Rev Microbiol 23: 269–354. 9439886 10.3109/10408419709115140

[51] Sirand-Pugnet P, Citti C, Barre A, Blanchard A (2007) Evolution of *Mollicutes*: down a bumpy road with twists and turns. Res Microbiol 158: 754–766. 18023150 10.1016/j.resmic.2007.09.007

[52] Razin S (2007) Molecular biology and genomics of *Mollicutes* . B Insectol 60: 101–103.

[53] Pinel N, Davidson SK, Stahl DA (2008) *Verminephrobacter eiseniae* gen. nov., sp nov., a nephridial symbiont of the earthworm *Eisenia foetida* (*Savigny*). Int J Syst Evol Micr 58: 2147–2157. 10.1099/ijs.0.65174-0 18768621

[54] Willems A, Falsen E, Pot B, Jantzen E, Hoste B, et al (1990) *Acidovorax*, a new genus for *Pseudomonas facilis*, *Pseudomonas delafieldii*, E. Falsen (Ef) group 13, Ef group 16, and several clinical isolates, with the species *Acidovorax facilis* comb. nov., *Acidovorax delafieldii* comb. nov., and *Acidovorax temperans* sp. nov. Int J Syst Bacteriol 40: 384–398. 2275854 10.1099/00207713-40-4-384

[55] Schulze R, Spring S, Amann R, I H, Ludwig W, et al (1999) Genotypic diversity of *Acidovorax* strains isolated from activated sludge and description of *Acidovorax defluvii sp nov* . Syst Appl Microbiol 22: 205–214. 10390871 10.1016/S0723-2020(99)80067-8

[56] Pandazis G (1931) Zur Frage der Bakteriensymbiose bei *Oligochaeten* . Zentralbl Bakteriol 440–453 p.

[57] Ní Chadhain SM, Schaefer JK, Crane S, Zylstra GJ, Barkay T (2006) Analysis of mercuric reductase (*merA*) gene diversity in an anaerobic mercury-contaminated sediment enrichment. Environ Microbiol 8: 1746–1752. 16958755 10.1111/j.1462-2920.2006.01114.x

[58] Rasmussen LD, Zawadsky C, Binnerup SJ, Øregaard G, Sørensen SJ, et al (2008) Cultivation of hard-to-culture subsurface mercury-resistant bacteria and discovery of new *merA* gene sequences. Appl Environ Microb 74: 3795–3803. 10.1128/AEM.00049-08 PMC2446565 18441111

[59] Robinson JB, Tuovinen OH (1984) Mechanisms of microbial resistance and detoxification of mercury and organomercury compounds-physiological, biochemical, and genetic analyses. Microbiol Rev 48: 95–124. 6377034 10.1128/mr.48.2.95-124.1984 PMC373215

[60] Barkay T, Miller SM, Summers AO (2003) Bacterial mercury resistance from atoms to ecosystems. FEMS Microbiol Rev 27: 355–384. 12829275 10.1016/S0168-6445(03)00046-9

[61] Barkay T, Kritee K, Boyd E, Geesey G (2010) A thermophilic bacterial origin and subsequent constraints by redox, light and salinity on the evolution of the microbial mercuric reductase. Environ Microbiol 12: 2904–2917. 20545753 10.1111/j.1462-2920.2010.02260.x

[62] Wiatrowski HA, Ward PM, Barkay T (2006) Novel reduction of mercury(II) by mercury-sensitive dissimilatory metal reducing bacteria. Environ Sci Technol 40: 6690–6696. 17144297 10.1021/es061046g

[63] Peretyazhko T, Charlet L, Muresan B, Kazimirov V, Cossa D (2006) Formation of dissolved gaseous mercury in a tropical lake (Petit-Saut reservoir, French Guiana). Sci Total Environ 364: 260–271. 16061273 10.1016/j.scitotenv.2005.06.016

[64] Oregaard G, Sorensen SJ (2007) High diversity of bacterial mercuric reductase genes from surface and sub-surface floodplain soil (Oak Ridge, USA). ISME J 1: 453–467. 18043664 10.1038/ismej.2007.56

[65] Spangler WJ, Spigarel.Jl, Rose JM, Miller HM (1973) Methylmercury-bacterial degradation in lake sediments. Science 180: 192–193. 17811660 10.1126/science.180.4082.192

[66] Walsh CT, Distefano MD, Moore MJ, Shewchuk LM, Verdine GL (1988) Molecular-basis of bacterial-resistance to organomercurial and inorganic mercuric-salts. Faseb J 2: 124–130. 3277886 10.1096/fasebj.2.2.3277886

[67] Sellers P, Kelly CA, Rudd JWM, MacHutchon AR (1996) Photodegradation of methylmercury in lakes. Nature 380: 694–697.

